# Cartilage Conduction Hearing Aids in Clinical Practice

**DOI:** 10.3390/audiolres13040045

**Published:** 2023-07-13

**Authors:** Tadashi Nishimura, Hiroshi Hosoi, Ryota Shimokura, Tadashi Kitahara

**Affiliations:** 1 Department of Otolaryngology-Head and Neck Surgery, Nara Medical University, 840 Shijo-cho, Kashihara 634-8522, Nara, Japan; tkitahara@naramed-u.ac.jp; 2 MBT (Medicine-Based Town) Institute, Nara Medical University, 840 Shijo-cho, Kashihara 634-8522, Nara, Japan; hosoi@naramed-u.ac.jp; 3 Graduate School of Engineering Science, Osaka University, D436, 1–3 Machikaneyama, Toyonaka 560-8531, Osaka, Japan; rshimo@sys.es.osaka-u.ac.jp

**Keywords:** bone conduction, cartilage conduction, hearing device, amplification, aural atresia, canal stenosis, conductive hearing loss, chronic otitis media

## Abstract

A relatively loud sound is audible when a vibrator is attached to the aural cartilage. This form of conduction is referred to as cartilage conduction (CC). In Japan, a new type of hearing aid has been developed using CC and has been available in clinical practice since 2017. A clinical study conducted prior to its launch demonstrated its benefits, particularly in patients with aural atresia who were unable to use air conduction hearing aids. Several studies have been published on the benefits of CC hearing aids since their introduction into clinical practice. Most of the patients included in these studies had canal stenosis or aural atresia, and the purchase rates of CC hearing aids in these patients were relatively high. However, the number of patients with canal-open ears was small, with overall poor results in the trials, with the exception of patients with continuous otorrhea. CC hearing aids are considered a good option for compensating for hearing loss in ears with canal stenosis or atresia in both bilateral and unilateral cases. However, CC hearing aids are not currently considered the first choice for patients with a canal-open ear.

## 1. Introduction

Sound is generally delivered to the ear via air conduction (AC) in conventional hearing aids. AC hearing aids amplify signals to help patients with various hearing losses. Unfortunately, some patients are unable to receive adequate benefits from AC hearing aids. For instance, in patients with aural atresia, hearing aids cannot be worn owing to anatomical issues or they receive inadequate benefits even if they can be worn [1]. In addition, continuous otorrhea prevents the use of hearing aids because they can prolong the inflammation, damage hearing aids, and obstruct the bore, thereby deteriorating the signal [2]. Bone conduction (BC) hearing aids have been considered as an alternative. In conventional BC hearing aids, a vibrator with static force is placed on the mastoid using a headband. BC hearing aids are effective in amplifying sound in the above-mentioned cases because sound is transmitted via BC [3,4,5]. In contrast, the fixed form of BC causes various problems, such as skin induration, long-continued depressions in the skin, and discomfort [3,4]. Furthermore, fixation with a headband is considered an esthetic disadvantage. Therefore, BC hearing aids are not preferred in patients who can use AC hearing aids without serious complications, and are rarely used in patients with unilateral aural atresia.

**Figure 1:**
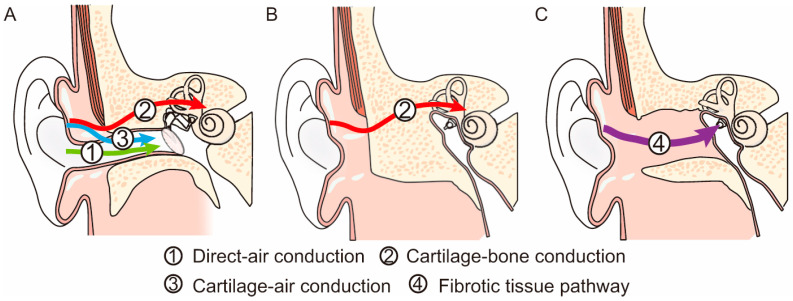
Difference in signal transmission in the normal ear (**A**), a bony atretic ear (**B**), and a fibrotic atretic ear with a fibrotic tissue pathway (FTP) (**C**).

When a vibrator is attached to the aural cartilage, hearing is significantly improved compared with that in the unattached condition. This phenomenon was confirmed by using a probe microphone [6]. Previous studies have demonstrated this improvement to be significant, particularly at low to middle frequencies [6,7,8,9]. This unique form of transmission is called cartilage conduction (CC) [10]. Figure 1A shows the predominant pathways theoretically assumed in CC [11,12]. The first pathway is direct AC. The vibrator radiates sound around it, which cannot be completely eliminated. This airborne sound travels through the ear canal to drive the eardrum and the ossicles. This pathway is considered an AC pathway. The second pathway is the cartilage–BC. Vibrations are delivered to the skull bone via the aural cartilage, and the vibrations of the skull bone are transmitted to the cochlea in the same manner as in BC. Mediation by the aural cartilage could deteriorate these signals. This pathway is considered the BC pathway. The third pathway is the cartilage–AC. The delivered vibrations of the cartilaginous portion of the ear canal generate airborne sounds in the ear canal. The cartilaginous portion of the ear canal functions as a movable plate during this process [13]. This third pathway is not the predominant signaling route in either AC or BC. However, the airborne sound level in the cartilage AC is considered to be larger than that in direct AC in CC. The differences in the elevations of the thresholds with the insertion of an earplug and the injection of water into the ear canal demonstrated a significant cartilage AC function [11,12,14,15]. Previous studies have concluded that CC varies in the transduction method from AC and BC.

## 2. Development of CC Hearing Aids

CC hearing aids are new hearing devices utilizing CC [16,17,18]. CC hearing aids were first developed in 2010 [16]. The characteristics of CC hearing aids are more similar to those of AC hearing aids than BC hearing aids because sound is finally transmitted to the cochlea via the eardrum and ossicles. In contrast to AC hearing aids, CC hearing aids deliver sounds to the aural cartilage as vibrations. In patients with aural atresia, skull vibrations are required to transmit sounds to the cochlea using any hearing device. Sound deterioration in CC is considerably lower than that in AC because it avoids the boundary between the air and the body during sound transmission. The vibrator of a CC hearing aid is placed on the aural cartilage without contact force, which is different from that of BC hearing aids. To fix it, the vibrator is inserted into the cavity or attached with double-sided tape. This fixation style can resolve the problems experienced in BC hearing aid use. In patients with continuous otorrhea, ear canal opening contributes to the continuous use of hearing devices. The vibrator of the CC hearing aid can be placed to keep the ear canal open, thereby contributing to ventilation; moreover, it is completely waterproof, which reduces the risk of damage to the vibrator. The audiological benefits of the prototype CC hearing aids were evaluated in a previous study to demonstrate their benefits, particularly in patients with aural atresia [19].

The initial prototype CC hearing aid was a box type, and the transducer was not compact [16,17]. Furthermore, a piezoelectric transducer was employed, which required a high-voltage battery for proper function. Therefore, using this prototype in clinical practice is challenging. A new electromagnetic transducer was developed as a CC hearing aid for clinical practice. It functions using the same battery used in commercially available AC hearing aids. This new transducer contributed to the miniaturization and production of a behind the ear (BTE)-style hearing aid. A clinical study was performed using the devised BTE CC hearing aids, mainly in patients with aural atresia [19]. Forty-one patients (21, 15, and 5 with bilateral aural atresia, unilateral aural atresia, and other conductive hearing loss, respectively) participated in the study. Most patients with bilateral aural atresia had used BC hearing aids before the trial. No significant differences were observed in the aided thresholds and speech recognition between the CC and BC hearing aids. After the trial, 20 patients with bilateral aural atresia continued to use the CC hearing aids. Nearly none of the patients with unilateral aural atresia used any hearing device. The functional gains obtained using the CC hearing aid were similar to those observed in patients with bilateral aural atresia. After the trial, 14 patients continued to use the CC hearing aids. A clinical study has demonstrated the effectiveness of CC hearing aids [19]; moreover, CC hearing aids were approved as new medical devices by the Ministry of Health, Labor, and Welfare in Japan and have been used in clinical practice in Japan since 2017.

## 3. Performance of CC Hearing Aids

**Figure 2:**
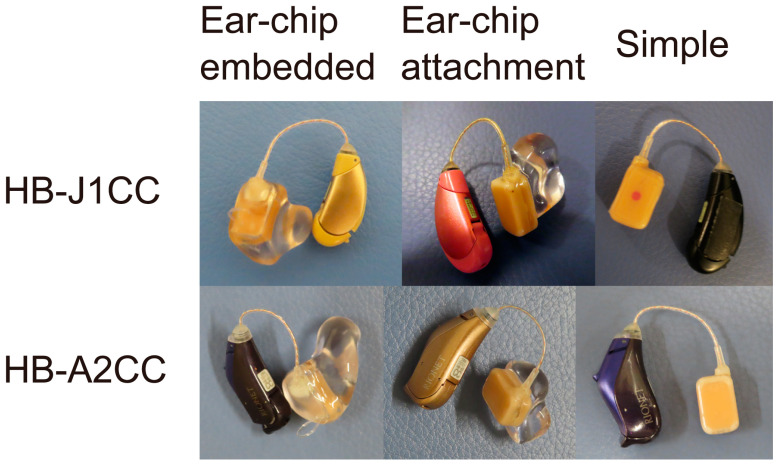
Two models of cartilage conduction hearing aids used in clinical practice: HB-J1CC (**upper**) and HB-A2CC (**lower**). Both models have three transducer types: ear-chip embedded (**left**), ear-chip attachment (**middle**), and simple vibrator (**right**).

Commercially available CC hearing aids are small BTE hearing aids, which were developed based on those used in clinical studies. The main body was designed based on that used in commercially available receiver-in-canal (RIC)-style AC hearing aids. The vibrator is connected to the main body with a wire that encapsulates the electrode within. Three types of vibrator units (ear-chip embedded, ear-chip attachment, and simple) are employed (Figure 2). The size and mass of the assembled transducer are 11.9 × 7.8 × 4.7 mm and 1.4 g, respectively. This type was chosen based on the ear condition. The ear chips are custom fitted, made based on ear impressions. Instead of taking an impression of the ear, computed tomography (CT) images can also be utilized for designing the vibrator [20]. Compared to the conventional process, the merits of the design using CT images are as follows: no risks related to taking the ear impression, advantage of understanding the shape of the ear in 3D, no physical transport or shipment of an ear impression, and CT images can be sent instantly via the internet. Therefore, CC hearing aids can be created without visiting the hospital. If a CT scan is performed for diagnosis or other purposes, the images can be used without additional risk. A previous study reported that the performance of a CT-based vibrator is not significantly inferior to that of an impression-based vibrator [20]. In contrast, the simple type is available for all ear conditions and can be prepared in advance; patients can try it quickly and unnecessary ear chip costs are also reduced. However, the simple type requires double-sided tape for fixation. Among the three types, the custom-fitted type is recommended for improved stability when the cavity of the fixation placement is sufficient to hold the transducer. A previous study [21] that investigated the differences in the purchase rates demonstrated a decreased purchase rate particularly in canal-open ears when a simple vibrator was used for the trial of the CC hearing aid.

Two CC hearing aid (HB-J1CC and HB-A2CC; Rion Co Ltd., Kokubunji, Japan) models are commercially available (Figure 2). The transducers used in the vibrator are identical. The functions of the two devices vary slightly. HB-A2CC is a later model that has been modified to reflect the feedback obtained from HB-J1CC users. Both devices were adjusted using fitting software. The gains, compression rates, and maximum output levels can be controlled. Linear amplification is utilized in patients with conduction hearing loss, such as those with aural atresia. The fitting software depicts the frequency responses on the screen; however, these simulated gains are not always equal to the actual values. Therefore, the real gains must be confirmed by measuring the unaided and aided thresholds. Both devices can manage feedback problems and directional modes. While only one program can be memorized for HB-J1CC, three programs can be used to switch memories for HB-A2CC. Furthermore, HB-A2CC can be connected via Bluetooth with an Android smartphone using an application and equipped with a child safety lock for the battery locker.

## 4. Benefits of CC Hearing Aids

CC hearing aids were newly devised and first launched in Japan in 2017. To date, no clinical data are available concerning CC. To determine the indications, a clinical survey was conducted in 2019. Nine medical institutions participated in the study, and 256 patients were registered [22]. In total, 113 and 143 patients had bilateral and unilateral hearing loss, respectively. Considering the previous results, CC hearing aids appear promising in patients with aural atresia. A total of 65 patients had bilaterally closed ears (aural atresia or severe stenosis), and 56 (86%) purchased CC hearing aids after fitting. This high purchase rate is consistent with the results of a previous clinical trial [22]. In addition to the atretic ear, it is also difficult to use AC hearing aids in patients with continuous otorrhea. Of nine patients with bilateral chronic continuous otorrhea, seven (78%) purchased CC hearing aids after fitting. The purchase rate was comparable to that of patients with bilaterally closed ears. In contrast, 27 patients with bilateral canal-open ears who could use AC hearing aids without difficulty tried CC hearing aids, and 10 patients (37%) purchased them after fitting. In the unilateral cases, 124 and 13 patients had closed and canal-open ears, respectively. After fitting, 97 patients (78%) with a unilateral closed ear purchased CC hearing aids, while 7 patients (54%) with a unilateral canal-open ear purchased them. The purchase rate for bilateral canal-open ear cases was significantly lower than those for bilateral and unilateral closed ear cases. Furthermore, seven patients with unilateral profound deafness tried CC hearing aids in their dead ear. They anticipated the effectiveness of the transcranial contralateral routing of signal (CROS) hearing aid to be similar to that of the bone anchored hearing aid (BAHA) for single-side deafness [5,23]. After the trial, four patients (57%) purchased hearing aids, indicating a significant benefit of CROS hearing aids in some patients. Thus, the clinical survey suggested that CC hearing aids are a good option not only for patients with closed ears, but also for those who have difficulties with the use of AC hearing aids.

In addition to the abovementioned clinical surveys, several medical institutions have reported the results of CC hearing aid fittings. Sakamoto et al. evaluated the benefits of CC hearing aids in children with unilateral congenital atretic ears and reported that the speech recognition scores improved in noisy environments as well as with the FM system [24]. The authors recommended FM systems and CC hearing aids for audiological management to improve speech recognition in children with unilateral aural atresia in classrooms. Akasaka et al. evaluated the benefits of CC hearing aids for speech perception in patients with unilateral aural atresia [25]. Speech recognition scores at low speech levels significantly improved in the aided atretic ear condition. They demonstrated that CC hearing aids in the unilateral atretic ear provided a diotic summation effect, which is considered a binaural hearing benefit.

Nishiyama et al. assessed the efficacy of CC hearing aids in adult patients with hearing loss and with various anatomical ear canal conditions to identify suitable candidates for CC hearing aids [26]. They categorized patients into three groups based on the anatomy of the ear canal: canal stenosis (or aural atresia), abnormal canal, and normal canal. Over 70% of the participants with canal stenosis purchased CC hearing aids, regardless of their AC hearing thresholds. In contrast, in the abnormal canal group, the purchase rates significantly depended on the AC hearing thresholds. The purchase rate of participants with mild hearing loss was higher than that of participants with severe hearing loss (85.71% vs. 20%). They concluded that patients with ear canal stenosis or atretic ears were the best candidates regardless of their hearing thresholds. Furthermore, they also reported the results of CC hearing aid fitting in children [27]. They fitted CC hearing aids in 48 ears of 42 patients. Forty of them were patients with canal stenosis and atresia. Overall, 72.92% of the participants made purchases after the trial. Additional tape compression was applied over the vibrator and the hearing improvement and adverse effects were assessed. An improvement in gains at low frequencies was observed; moreover, application of the additional compression tape resulted in no side effects. The authors concluded that CC hearing aids are a good option for hearing improvement in children with canal stenosis or aural atresia who cannot use AC hearing aids.

Takai et al. fitted CC hearing aids in 41 patients, 19 (65.9%) of whom purchased them after the trial [28]. They compared the clinical characteristics of the patients who purchased and did not purchase the hearing aids, and found that the rate of congenital canal stenosis or aural atresia was significantly higher in purchased cases than in the non-purchased cases. They also found that those who decided to purchase CC hearing aids showed better hearing thresholds at high frequencies for both AC and BC as well as for aided thresholds when using CC hearing aids.

Several studies have reported the benefits of CC hearing aids in clinical practice in Japan. Most patients who attempted to use CC hearing aids experienced canal stenosis or aural atresia. The audiological benefits in these cases were significant, and the reported purchase rates were good. Patients with unilateral canal stenosis or aural atresia rarely used amplification devices before the CC hearing aid trial. However, the purchase rates of CC hearing aids in these cases were comparable to those in bilateral cases [22]. No significant adverse effects were reported, which probably contributes to the promotion of the use of CC hearing aids in unilateral cases, unlike other hearing devices. Thus, CC hearing aids are considered a good option for compensating for hearing loss in ears with canal stenosis or aural atresia in both bilateral and unilateral cases. However, current CC hearing aids are not considered the first choice for cases with a canal-open ear. Nevertheless, they can provide significant benefits in specific cases such as continuous otorrhea. The indications for the CC hearing aids in these cases are limited. However, the fitting cases in previous studies were not sufficient to draw this conclusion. Further studies are warranted to clarify the indications in canal-open ears.

## 5. Clinical Studies in Countries Other Than Japan

CC hearing aids are currently used solely in Japan in clinical practice and cannot be purchased in other countries. However, clinical studies have already been conducted in two countries. In Indonesia, Suwento et al. measured the benefits of CC hearing aids in ten patients (aged <20 years) with microtia and aural atresia whose hearing dysfunction did not improve after ear reconstruction surgery [29]. They found a significant difference between unaided and aided thresholds. Speech recognition thresholds and speech discrimination levels were also significantly improved with the use of CC hearing device. Almost all the parents reported satisfaction with the performance of the CC hearing aids upon daily communication with their children.

Considering the effectiveness of CC hearing aids in the atretic ear, the difference between the benefits of BC devices and CC hearing aids is an interesting subject. In the United States, Nairn et al. compared the benefits of BC devices (BAHA 5, BAHA 5 power (Cochlear Limited, Sydney, Australia) and Ponto 4 (Oticon Medical, Smørum, Denmark)) and CC hearing aids (HB-A2CC) using a crossover study design [30]. Sixteen adults (19 ears) with congenital aural atresia or overclosed ear canals who previously underwent BC device implantation participated in the study. The mean aided pure tone averages with the BC device and CC hearing aids were 27 and 32 dB, respectively, and the mean functional gains were 54 and 49 dB, respectively. Significant differences were observed between them. Regarding speech perception, the mean consonant-nucleus-consonant scores with the BC device were 90% (best aided) and 80% (aided ear isolated), and those with the CC hearing aid were 86% and 76%, respectively. The mean AzBio scores were 90% (quiet), 77% (+10 dB signal to noise ratio (SNR)), and 52% (+5 dB SNR) when isolating the BC device ear, and 90%, 73%, and 41% when isolating the CC hearing aid ear. No difference in speech scores achieved statistical significance, except for AzBio isolated from the aided ear in the 15 dB SNR condition, which favored the BC device. They concluded that pure-tone audiometric outcomes with the BC device demonstrated a small advantage over the CC hearing aid, with the difference being driven mainly by high-frequency responses. Speech outcomes were equivalent, except for the 15dB SNR condition. Regarding the differences between BC devices and CC hearing aids, Nishiyama et al. compared the benefits of the BAHA, CC hearing aids, and ADHEAR (MED-EL, Innsbruck, Austria) [31]. They reported data from six patients who underwent comparative trials. The functional gains for the BAHA and CC hearing aids improved compared with those of the ADHEAR in Japan. In contrast, no clear tendency was observed among the three devices in a quality of life evaluation. They indicated the need for comparative trials and consultations when selecting a device.

## 6. Signal Transmission Pathway to the Cochlea in Atretic Ears

CC hearing aids are effective in the atretic ear, and most patients purchased them after the trial. From the viewpoint of signal transmission, the pathway to the cochlea in the atretic ear is quite different from that in the normal ear. The cartilage–AC predominantly contributes to hearing in normal ears. However, both direct and cartilage–AC pathways are absent in the atretic ear. Theoretically, the signal transmission pathway should include the skull bone in the atretic ear for conduction. Thus, the predominant pathway to the cochlea switches from cartilage–AC to cartilage–BC in the atretic ear (Figure 1B). The transmission efficacy may decrease in the atretic ear based on the difference in contribution to the threshold between cartilage–AC and BC in the normal ear. Compared with the vibrator placed on the mastoid, the delivered vibrations could deteriorate because they are delivered to the skull bone via the cartilaginous tissues. A previous study compared the thresholds of a vibrator on the aural cartilage and those on the mastoid (cartilage and mastoid stimulation conditions) [32]. A previous study demonstrated the thresholds at low frequencies to be significantly better in the cartilage stimulation condition, and that no difference was present in the thresholds at high frequencies, implying that the fixation placement had no negative effect. Furthermore, the static force is important for signal transmission in BC [33,34]. In a normal ear, the sound pressure level in the ear canal produced by CC is also influenced by static forces [6]. It increases as a function of the static force. One of the greatest benefits of CC hearing aids is their comfort while wearing them, which is attributed to their fixation style. CC hearing aids are typically used without static force; this fixation style could negatively affect signal transmission.

## 7. Benefits of CC in Atretic Ears with Fibrotic Pathways

An absent ear canal can occur due to congenital anomalies, as well as acquired factors such as inflammation, injury, and surgical treatment. In the latter case, the ear canal is usually closed with no bony tissue, and signals delivered to the cartilaginous tissue travel via the fibrotic tissues to drive the remaining ossicles when fibrotic tissues are connected to the remaining ossicles (fibrotic tissue pathway) (Figure 1C). Signals are effectively transmitted in cases involving the fibrotic tissue pathway because vibrations of the large-mass skull bone are not mandatory in this transmission. A previous study compared the CC and BC thresholds in patients with and without fibrotic tissue pathways [35]. The findings demonstrated an improvement in the thresholds of the fibrotic pathway, and the benefits became more significant as the frequency decreased. In another study, the thresholds in atretic ears with a fibrotic pathway significantly improved by approximately 20 dB at frequencies below 1000 Hz when the transducer was placed on the aural cartilage [32]. No differences were observed in the thresholds at frequencies above 2000 Hz. The threshold difference between cartilage and mastoid stimulations increases in the atretic ear via a fibrotic pathway. These findings imply that the audiological benefits of CC hearing aids are greater in the atretic ear via the fibrotic pathway. Komune et al. used CC hearing aids to manage residual hearing following lateral temporal bone resection in patients with temporal bone malignancies [36]. The hearing outcomes of patients who have undergone external auditory meatus reconstruction vary widely. They used CC hearing aids instead of ear canal reconstruction to compensate for the hearing loss. The performance of CC hearing aids revealed individual variations. They found that the difference between the aided and BC thresholds increased as the distance between the bone and cartilage increased. Although there is still room for improvement in the surgical techniques, they concluded that CC hearing aids provide noninvasive postoperative hearing compensation following lateral bone resection.

## 8. Sound Localization in Bilateral Atretic Ears

One benefit of binaural hearing is sound localization. Patients with bilateral aural atresia often exhibit poor sound localization due to BC features (low intracranial attenuation). However, most patients using CC hearing aids have reported improvements. Nishimura et al. evaluated sound localization by using eight loudspeakers positioned in a full-circle at 45 degree intervals in patients with bilateral aural atresia [37]. They compared the results of hearing unaided, aided by previously used hearing aids (AC or BC hearing aids), and aided by CC hearing aids. The ability to distinguish sounds originating from the left or right side for participants aided by CC hearing aids was significantly better than that for the other conditions. The transmission pathway to the cochlea involves the skull in all cases. Therefore, another cue that distinguishes between the left and right may function in the CC. They hypothesized the involvement of another mechanism, such as the contribution of the vibration sensation. The vibrator on the aural cartilage vibrates for sound transmission, and this vibration may induce both the auditory and somatic sense [37]. This somatic sense could provide a cue for differentiating between the left and right sides. BC hearing aids transmit sounds transcutaneously. However, the vibrator is tightly attached to the bone in the BC hearing aid; thus, the somatic sense may become damaged and dull. Conversely, the vibrators of the CC hearing aids were attached without high contact pressure, and the somatic sensation was maintained. However, the contribution of the somatic sense to sound localization remains to be clarified, and further studies are warranted.

Kitama et al. measured the sound localization in patients with unilateral atretic ears using a CC hearing aid, BAHA, and ADHEAR on the atretic ear. Compared with the un-aided condition, no significant improvement was observed in any of the three aided conditions [31]. However, the comparison was provided for only one patient. Thus, a firm conclusion could not be drawn regarding the effect of CC hearing aids on sound localization in patients with unilateral atretic ears.

## 9. Auricular Prosthesis

Esthetic problems are considered a disadvantage of hearing devices. Compared to BC hearing aids, CC devices are smaller and a headband is not required for fixation. Unfortunately, CC hearing aids are not devoid of esthetic problems, despite the esthetic advantages in comparison with BC hearing aids. Congenital aural atresia is often accompanied by microtia, which also causes esthetic problems. Nishiyama et al. developed an auricular prosthesis incorporating a cartilage conduction hearing aid (APiCHA) to achieve the challenging goal of simultaneously improving both esthetic problems [38]. Compared with the CC hearing aid alone, the functional gain was approximately 2 dB lower at high frequencies from 1 kHz and above, and approximately 2 dB higher at high frequencies from 900 Hz when the CC hearing aid was used with the APiCHA. They reported that the combined use of the APiCHA and CC hearing aids can be considered a noninvasive and clinically applicable treatment option to achieve both esthetic and auditory improvements for microtia.

## 10. Conclusions

CC hearing aids were launched in Japan in 2017. The number of clinical cases in which this new device has been used has increased greatly, with several studies reporting its benefits. According to the results, CC hearing aids are considered a good option for compensating for hearing loss in ears with canal stenosis or aural atresia in both bilateral and unilateral cases. However, CC hearing aids are not currently considered the first choice in patients with a canal-open ear. Nevertheless, they can provide significant benefits in specific cases, such as continuous otorrhea. Further studies are warranted to clarify the indications for use in canal-open ears.

## Data Availability

Not applicable.
